# Effect of methotrexate and anti-TNF on Epstein-Barr virus T-cell response and viral load in patients with rheumatoid arthritis or spondylarthropathies

**DOI:** 10.1186/ar2708

**Published:** 2009-05-26

**Authors:** Corinne Miceli-Richard, Nicolas Gestermann, Corinne Amiel, Jérémie Sellam, Marc Ittah, Stephan Pavy, Alejandra Urrutia, Isabelle Girauld, Guislaine Carcelain, Alain Venet, Xavier Mariette

**Affiliations:** 1 Rhumatologie, Hôpital Bicêtre, Assistance Publique-Hôpitaux de Paris (AP-HP), 78 rue du Général Leclerc, 94275 Le Kremlin Bicêtre, France; 2 Institut Pour la Santé et la Recherche Médicale (INSERM) U 802, Université Paris-Sud 11, 64 rue Gabriel Péri, 94275 Le Kremlin Bicêtre, France; 3 Virologie, Hôpital Tenon, AP-HP, 4 rue de la Chine, 75020 Paris, France; 4 INSERM U543, Hôpital La Pitié Salpétrière, AP-HP, 47 Boulevard de l'Hôpital, 75013 Paris, France

## Abstract

**Introduction:**

There is a suspicion of increased risk of Epstein-Barr virus (EBV)-associated lymphoproliferations in patients with inflammatory arthritides receiving immunosuppressive drugs. We investigated the EBV load and EBV-specific T-cell response in patients treated with methotrexate (MTX) or anti-TNF therapy.

**Methods:**

Data for patients with rheumatoid arthritis (RA) (n = 58) or spondylarthropathy (SpA) (n = 28) were analyzed at baseline in comparison with controls (n = 22) and after 3 months of MTX or anti-TNF therapy for EBV load and EBV-specific IFNγ-producing T cells in response to EBV latent-cycle and lytic-cycle peptides.

**Results:**

The EBV load and the number of IFNγ-producing T-cells after peptide stimulation were not significantly different between groups at baseline (*P *= 0.61 and *P *= 0.89, respectively). The EBV load was not significantly modified by treatment, for RA with MTX (*P *= 0.74) or anti-TNF therapy (*P *= 0.94) or for SpA with anti-TNF therapy (*P *= 1.00). The number of EBV-specific T cells was not significantly modified by treatment, for RA with MTX (*P *= 0.58) or anti-TNF drugs (*P *= 0.19) or for SpA with anti-TNF therapy (*P *= 0.39). For all patients, the EBV load and EBV-specific T cells were significantly correlated (*P *= 0.017; *R *= 0.21). For most patients, short-term exposure (3 months) to MTX or anti-TNF did not alter the EBV load or EBV-specific T-cell response but two patients had discordant evolution.

**Conclusions:**

These data are reassuring and suggest there is no short-term defect in EBV-immune surveillance in patients receiving MTX or anti-TNF drugs. However, in these patients, long term follow-up of EBV-specific T-cell response is necessary and the role of non-EBV-related mechanisms of lymphomagenesis is not excluded.

## Introduction

Rheumatoid arthritis (RA) is associated with a twofold increase of non-Hodgkin's lymphoma [1] and a threefold increase of Hodgkin's lymphoma [2]. The effect of immunosuppressive drugs on the risk of lymphoma is debated. Most recent studies did not find an overall increased risk of non-Hodgkin's lymphoma in RA patients treated with methotrexate (MTX). Several reports, however, showed that MTX can rarely induce Epstein-Barr virus (EBV)-associated lymphoproliferation regressive after withdrawal of the drug [3,4].

Recent concerns about possible treatment effects and lymphoma have focused on anti-TNF drugs because of their profound immunoregulatory effect. A recent meta-analysis of randomized controlled trials of infliximab and adalimumab identified 10 cases of lymphoma (four cases in the randomized phase of the trials and six cases in the extension phase) in the treated groups (3,493 patient-years) and none in the placebo groups (1,512 patient-years) [5]. Inflammatory activity of the underlying disease is the main risk factor of lymphoma in RA [6], however, and anti-TNF therapy is used for patients with the most active disease. Results for three large cohorts of RA patients did not reveal any increased risk of lymphoma in RA patients receiving anti-TNF drugs versus RA patients receiving classical disease-modifying anti-rheumatic drugs (DMARDs). In most of these cohorts, however, increased risk of lymphoma persisted as compared with that in the general population [7-9].

Cases of EBV-associated lymphoproliferation that regressed after withdrawal of MTX have been described [3,4]. Case reports of lymphoma associated or not with EBV, treated with anti-TNF drugs and regressing after withdrawal of therapy have also been reported [10,11]. These cases may mimic post-transplant lymphoproliferative disorder, a severe complication of EBV reactivation linked to impaired EBV control by CD8 T cells and arising in allograft recipients receiving immunosuppressive drugs [12].

Taken together, such data provide reliable arguments to investigate a potential EBV reactivation during MTX and/or TNFα antagonist therapy as a possible first step of lymphoma induction. During primary EBV infection, specific cytotoxic CD8^+ ^T cells expand and recognize epitopes from lytic-cycle antigens and, to a lesser extent, from latent-cycle antigens. A small population of EBV-specific memory CD8^+ ^T cells further persists [13] and plays a crucial role in the control of persistent EBV infection [14]. An impaired EBV-specific T-cell response could constitute one of the first steps of lymphoma induction with immunosuppressive drug therapy.

The present study aimed to determine the EBV viral load and the specific effector CD8^+ ^T-cell response against EBV antigens in patients with RA and spondylarthropathy (SpA) receiving MTX or anti-TNF drugs, to shed some light on a possible impaired EBV-specific T-cell response as the triggering mechanism of lymphomagenesis in this population.

## Materials and methods

### Study population

All studied subjects were seropositive for EBV. The present study consisted of two parts. In the cross-sectional first part of the study we investigated EBV-specific IFNγ-producing T cells at baseline (week 0) in 87 patients: 32 MTX naïve RA patients (mean age 60 ± 16 years, mean duration of disease 4.5 ± 6.6 years), 27 patients with RA receiving MTX who were not responders to the drug (mean age 53 ± 11 years, mean duration of disease 9.5 ± 10.5 years) and 28 patients with SpA (14 not receiving DMARDs and 14 receiving MTX; mean age 36 ± 11 years, mean duration of disease 9.6 ± 9.7 years). Patients with RA fulfilled the 1987 American College of Rheumatology criteria [15] and those with SpA fulfilled the European Spondylarthropathy Study Group criteria [16]. All RA patients were rheumatoid factor positive and/or anti-cyclic citrullinated peptide positive. The Disease Activity Score for 28 joints was 4.8 ± 1.2 in naïve RA patients and was 5.6 ± 1.2 in RA patients who were nonresponders to MTX. The Bath Ankylosing Spondylitis Disease Activity Index score [17] was 55 ± 22 in SpA patients. The control group comprised 22 patients with mechanic radiculopathic conditions (mean age 47 ± 15 years).

From the 87 patients included in the cross-sectional part of the study, 62 underwent the second longitudinal part of the study for EBV-specific IFNγ-producing T cells after 3 months (week 12) of MTX or anti-TNF treatment. Forty patients (21 SpA and 19 RA) received anti-TNF drugs. All RA patients and 10/21 SpA patients had anti-TNF + MTX. Twenty-two MTX naive RA patients received MTX. EBV viral load data were also available for 67 patients and 15 control individuals at week 0, and for 52 patients at week 12.

The present study was performed with approval of the local ethics committee (CPP Ile de France 7), and informed consent was obtained from all study participants.

### Isolation of peripheral blood mononuclear cells

Peripheral blood mononuclear cells (PBMCs) were isolated by density gradient centrifugation using Ficoll-Hypaque 1.107 (Biochrom, Berlin, Germany). The PBMCs were then frozen in FCS containing 10% dimethyl sulfoxide (Sigma, Saint-Quentin Fallavier, France) and stored in liquid nitrogen until use.

### Epstein-Barr virus peptides

A set of 39 9-mer latent-cycle peptides was used, corresponding to known human leukocyte antigen (HLA) class I-restricted cytotoxic T lymphocyte epitopes. Considering that the HLA status of our study patients and control individuals was unknown, these peptides were chosen as being recognized by a broad range of class I molecules [18]. The latent-cycle peptides used were immunodominant sequences from EBNA1, EBNA3A, EBNA3B, EBNA3C and LMP2 already tested in four different laboratories [18]. Lytic-cycle EBV antigens were represented by a BMLF 9-mer peptide and a collection of 47 overlapping 15-mer lytic-cycle peptides spanning the entire sequence of BZLF1 protein. The BMLF 9-mer is a peptide from the replicative phase of EBV previously reported to be an immunodominant HLA-A2-restricted epitope [19,20] (Table 1). Lyophilized peptides were dissolved in sterile water supplemented with 10% dimethyl sulfoxide at 40 μg/ml and were stored at -20°C. For peptide pulsing, target cells were incubated with peptides (final concentration 2 μg/ml). Individual responses to latent-cycle peptides and lytic-cycle peptides were summed and analyzed as a whole, and were also analyzed separately.

**Table 1:** Human leukocyte antigen class I-restricted cytotoxic T-lymphocyte Epstein-Barr virus epitopes

Human leukocyte antigen	Protein	Epitope position	Epitope sequence
A2	EBNA3A	596 to 604	SVRDRLARL
A2.01	EBNA3C	284 to 293	LLDFVRFMGV
A2.01	LMP2	329 to 337	LLWTLVVLL
A2.01	LMP2	426 to 434	CLGGLLTMV
A2.01	BMLF1	280 to 288	GLCTLVAML
A2.06	LMP2	453 to 461	LTAGFLIFL
A3	EBNA3A	603 to 611	RLRAEAQVK
A11	EBNA3B	399 to 408	AVFDRKSDAK
A11	EBNA3B	416 to 424	IVTDVSVIK
A11	LMP2	340 to 350	SSCSSCPLSKI
A23	LMP2	131 to 139	PYLFWLAAI
A24	EBNA3A	246 to 253	LYSIFFDY
A24	LMP2	419 to 427	TYGPVFMCL
A24.02	EBNA3B	217 to 225	TYSAGIVKI
A25	LMP2	442 to 451	VMSNTLLSAW
A29	EBNA3A	491 to 499	VFSDGRVAC
A30.02	EBNA3A	176 to 184	AYSSWMYSY
B7	EBNA3A	502 to 510	GPAPAGPIV
B7	EBNA3A	379 to 387	RPPIFIRRL
B7	EBNA3C	881 to 889	QPRAPIRPI
B8	EBNA3A	158 to 166	QAKWRLQTL
B8	EBNA3A	325 to 333	FLRGRAYGL
B8	BZLF1	190 to 197	RAKFKQLL
B27.02	EBNA3B	244 to 254	RRARSLSAERY
B27.02/.04/.05	EBNA3C	258 to 266	RRIYDLIEL
B27.04	LMP2	236 to 244	RRRWRRLTV
B27.05	EBNA3B	149 to 157	HRCQAIRKK
B27.05	EBNA3C	249 to 258	LRGKWQRRYR
B27.05	EBNA3C	343 to 351	FRKAQIQGL
B35	EBNA3A	458 to 466	YPLHEQHGM
B35	EBNA3B	488 to 496	AVLLHEESM
B35	BZLF1		EPLPQGQLTAY
B35.01	EBNA1	407 to 417	HPVGEADYFEY
B39	EBNA3C	271 to 278	HHIWQNLL
B44	EBNA3B	567 to 666	VEITPYKPTW
B44.02	EBNA3C	281 to 290	EENLLDFVRF
B44.02	EBNA3C	335 to 343	KEHVIQNAF
B44.03	EBNA3C	163 to 171	EGGVGWRHW
B60	LMP2	200 to 208	IEDPPFNSL
B62	EBNA3A	406 to 414	LEKARGSTY
B62	EBNA3B	831 to 839	GQGGSPTAM
B62	EBNA3C	213 to 222	QNGALAINTF

### ELISPOT assay

The ELISPOT-IFNγ assay was used to determine the frequency of T cells that produced IFNγ in response to a brief exposure to EBV antigens, as previously published [21]. Briefly, nitrocellulose ELISPOT plates (Millipore, Guyancourt, France) were coated with anti-IFNγ antibody (1 μg/ml, 100 μl/well in PBS; 1-D1K; Mabtech, Sophia Antipolis, France). PBMCs were added in duplicate wells at 10^5 ^cells per well with 2 μg/ml peptide. The second biotinylated anti-IFNγ monoclonal antibody was then added (7-B6-biotin; Mabtech) and IFNγ secreting cells were revealed with an enzymatic reaction with streptavidin-conjugated alkaline phosphatase (Sigma-Aldrich, Saint-Quentin Fallavier, France).

The number of specific T-cell responders per 10^6 ^PBMCs was calculated after subtraction of the background, which corresponded to the mean value of IFNγ spots associated with nonstimulated PBMCs (PBMCs in the presence of medium alone). Results were expressed as spot-forming cells (SFCs) per 10^6 ^PBMCs and were calculated for each pool of peptides as follows:

SFCs/10^6^ PBMCs = 10 x (mean SFCs/10^5^ cells from two antigen-stimulated wells - mean SFC/10^5^ cells from four unstimulated wells).

Results were presented as the individual response to the set of latent-cycle peptides (9-mer peptides), to the set of lytic-cycle peptides (BMLF 9-mer peptide added with the 15-mer lytic-cycle peptides) or to both sets.

Wells were counted as positive if they contained at least 50 SFCs/10^6 ^PBMCs and exhibited at least twofold the mean value of the background (per million PBMC). The median number of IFNγ-producing PBMCs in the presence of medium alone (background) was zero spots/well (range 0 to 4).

### Epstein-Barr virus load in peripheral blood mononuclear cells

The level of EBV DNA copies in PBMCs was measured by Taqman real-time quantitative PCR as previously described [22]. For each quantification, 500,000 to 10^6 ^PBMCs were thawed and DNA extractions were further performed. The PCR primers were selected to amplify a 121 bp product in the thymidine kinase gene. A pcDNA 3.1 vector (Invitrogen, Groningen, the Netherlands) containing one copy of the EBV target region was used as standard for EBV quantification. The level of albumin DNA copies in PBMC samples estimated by real-time PCR was used as the endogenous reference to normalize the variations in PBMC number or DNA extraction. All standard dilutions, control samples and PBMC samples were run in parallel and in duplicate for EBV and albumin DNA quantifications. The normalized value of the cell-associated EBV DNA load corresponding to the ratio EBV average copy number/albumin average copy number × 2.10^6 ^was finally expressed as the number of EBV DNA copies per 10^6 ^PBMC.

### Statistical analysis

Results are given as the percentage of patients with positive EBV T-cell response, as well as the mean response ± standard deviation. Statistical analyses involved use of StatView 5.0 (Abacus Concepts, Berkeley, CA, USA). Nonparametric tests were used. Comparisons between groups involved the Kruskal-Wallis test. Cross-sectional comparison of EBV T spots or the EBV copy number distribution involved the Mann-Whitney rank-sum test. Longitudinal comparison of EBV T spots or the EBV copy number between week 0 and week 12 involved the Wilcoxon test. Correlation studies involved Spearman's correlation. *P *< 0.05 was considered statistically significant.

## Results

### Cross-sectional study

#### Epstein-Barr virus load in peripheral blood mononuclear cells

**Figure 1:**
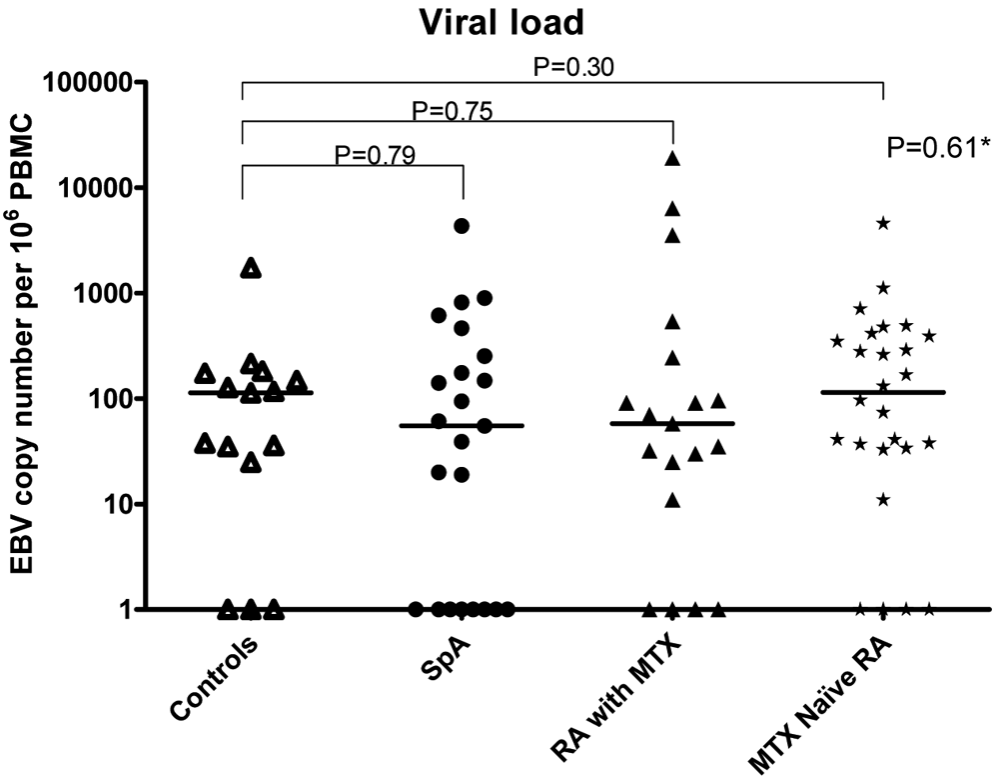
Epstein-Barr virus load in peripheral blood mononuclear cells in the cross-sectional study. Epstein-Barr virus (EBV) load distribution in control individuals (n = 15), spondylarthropathy (SpA) patients (n = 23), rheumatoid arthritis (RA) patients receiving methotrexate (MTX) (n = 18) and RA patients not receiving disease-modifying anti-rheumatic drug therapy (n = 26). Mean values of EBV viral load are represented by a black line. *Kruskall-Wallis test. PBMC, peripheral blood mononuclear cell.

The proportion of patients with positive EBV viral load did not differ among groups (control individuals, 80%; SpA patients, 65%; RA patients with MTX, 79%; and RA patients without DMARD treatment, 85% (*P *= 0.42, chi-square test)), nor did they differ when considering the distribution of all viral loads in the four groups of patients (*P *= 0.61, Kruskal-Wallis test) (Figure 1). Likewise, control individuals did not differ from any other group in viral load (Mann-Whitney test). The mean (± standard deviation) viral loads in each group were as follows: control individuals, 197 ± 433 copies/10^6 ^cells; SpA patients, 353 ± 905 copies/10^6 ^cells; RA patients with MTX, 1,596 ± 4,533 copies/10^6 ^cells; and MTX naïve RA patients, 387 ± 893 copies/10^6 ^cells. The median viral loads were 113 for control individuals, 55 for SpA patients, 58 RA patients with MTX, and 114 for MTX naïve RA patients.

We found no significant correlation between the EBV viral load and disease activity (Disease Activity Score for 28 joints for RA patients, *P *= 0.54; Bath Ankylosing Spondylitis Disease Activity Index for SpA patients, *P *= 0.84) or disease duration (*P *= 0.29).

#### Epstein-Barr virus-specific IFNγ-producing T cells

**Figure 2:**
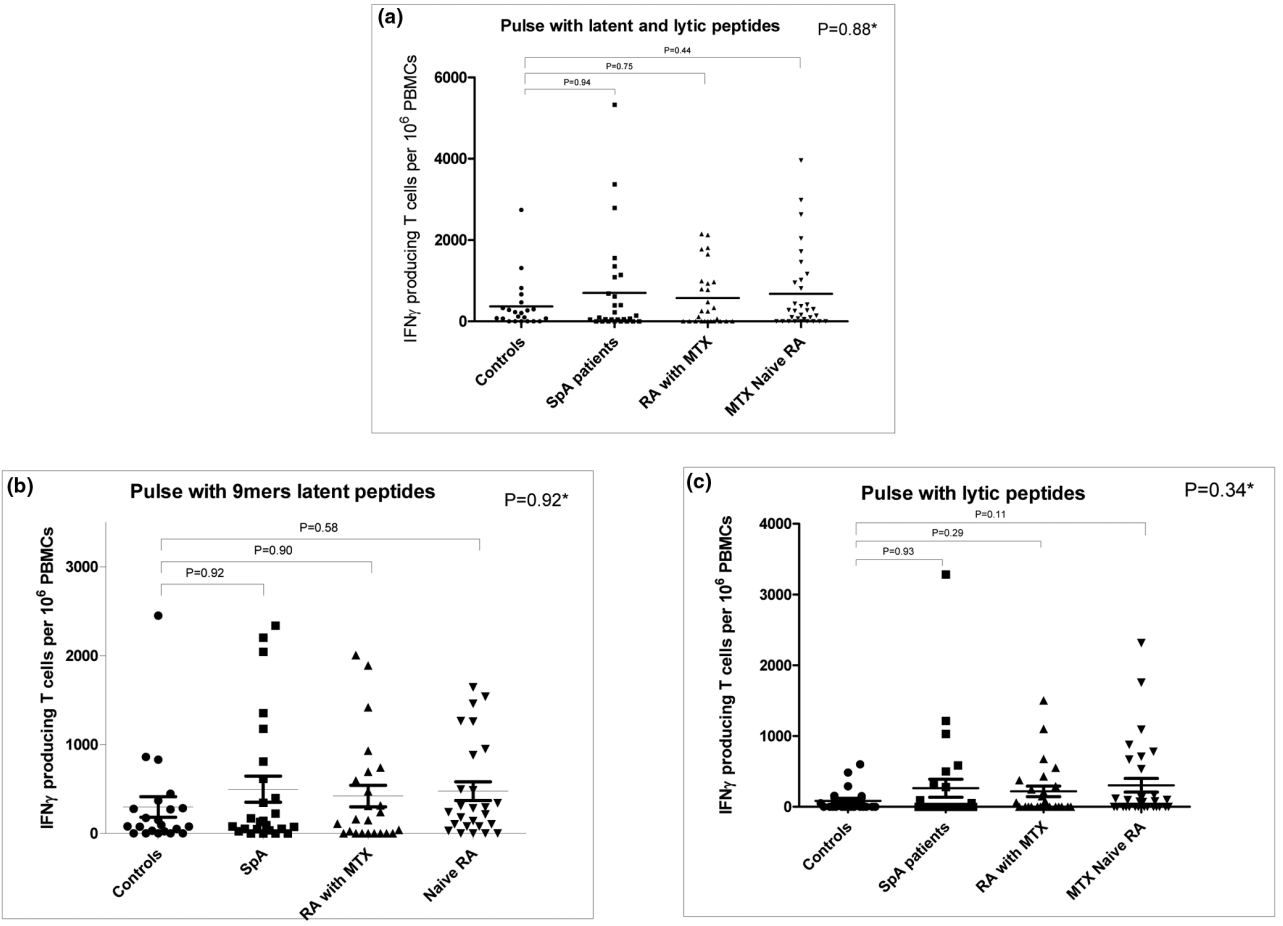
Epstein-Barr virus-specific IFNγ-producing T cells. Number of IFNγ-producing T cells per 10^6 ^peripheral blood mononuclear cells (PBMCs). **(a) **After pulsing with the full set of Epstein-Barr virus peptides. **(b) **After pulsing with latent-cycle peptides. **(c) **After pulsing with lytic-cycle peptides. Mean IFNγ-producing T cells per 10^6 ^PBMCs are represented by a black line. *Kruskall-Wallis test. MTX, methotrexate; RA, rheumatoid arthritis; SpA, spondylarthropathy.

The proportion of patients with positive EBV-specific IFNγ-producing T cells did not differ among groups (control individuals, 73%; SpA patients, 71%; RA patients with MTX, 59%; and RA patients without DMARD treatment, 72% (*P *= 0.68, chi-square test)) (Figure 2a). No significant differences were observed between groups when considering T-cell responses to the whole set of peptides (*P *= 0.86) or restricted to latent peptides (*P *= 0.92) or lytic peptides (*P *= 0.34) (Kruskal-Wallis test) (Figure 2b, c). The control group did not differ from each other treatment group either when considering pulses with the whole set of peptides, or when considering pulses with latent or lytic peptides (Mann-Whitney test) (Figure 2a to 2c).

We found no significant correlation between the number of EBV-specific IFNγ-producing T cells and disease activity (Disease Activity Score for 28 joints for RA patients (n = 64), *P *= 0.32; Bath Ankylosing Spondylitis Disease Activity Index for SpA patients (n = 21), *P *= 0.47) or disease duration (n = 88, *P *= 0.40).

### Longitudinal study

#### Epstein-Barr virus load in peripheral blood mononuclear cells

**Figure 3:**
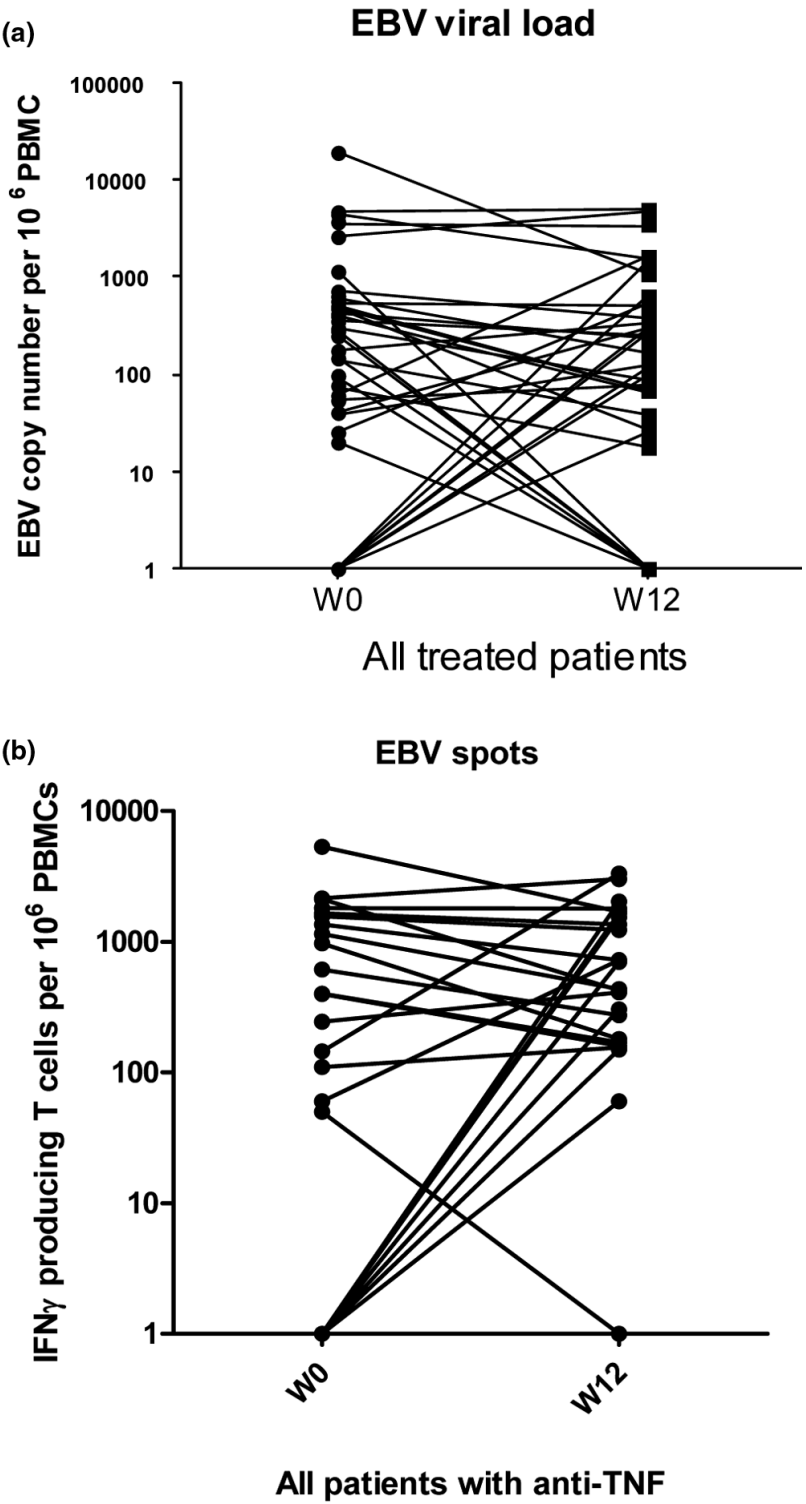
Epstein-Barr virus load in peripheral blood mononuclear cells in the longitudinal study. **(a) **Epstein-Barr virus (EBV) load between week 0 (W0) and week 12 (W12) for all treated patients (n = 42). **(b) **EBV-specific IFNγ-producing T cells per 10^6 ^peripheral blood mononuclear cells between W0 and W12 for all patients receiving anti-TNF drugs (n = 40). PBMCs, peripheral blood mononuclear cells.

When pooling all treatment groups, longitudinal observation of the EBV viral load showed no significant change between baseline (week 0) and week 12 (*P *= 0.33) (Wilcoxon test) (Figure 3a). Similar results were obtained when analyzing each treatment group longitudinally: SpA patients receiving anti-TNF drugs (*P *= 1.00), RA patients receiving anti-TNF drugs (*P *= 0.94) and RA patients receiving MTX (*P *= 0.74). Patients receiving anti-TNF drugs showed no difference in EBV viral load according to the class of TNF used: monoclonal antibody (infliximab and adalimumab) (n = 9, *P *= 0.31) or soluble receptor (etanercept) (n = 18, *P *= 0.63).

#### Epstein-Barr virus-specific IFNγ-producing T cells

In response to the full set of peptides, the number of IFNγ-producing cells was not significantly modified by immunosuppressive treatment (SpA patients receiving anti-TNF drugs (n = 21), *P *= 0.39; RA patients receiving anti-TNF drugs (n = 19), *P *= 0.19; RA patients receiving MTX (n = 22), *P *= 0.58) (Figure 3b), nor was the number of EBV-specific IFNγ-producing T cells modified when considering each set of peptides (latent-cycle peptides or lytic-cycle peptides) (data not shown). Among patients treated with TNF blockers, there was no difference according to the class of molecule used: monoclonal antibody (infliximab and adalimumab) (n = 16, *P *= 0.74) or soluble receptor (etanercept) (n = 24, *P *= 0.92).

### Correlation between Epstein-Barr virus load and T spots

**Figure 4:**
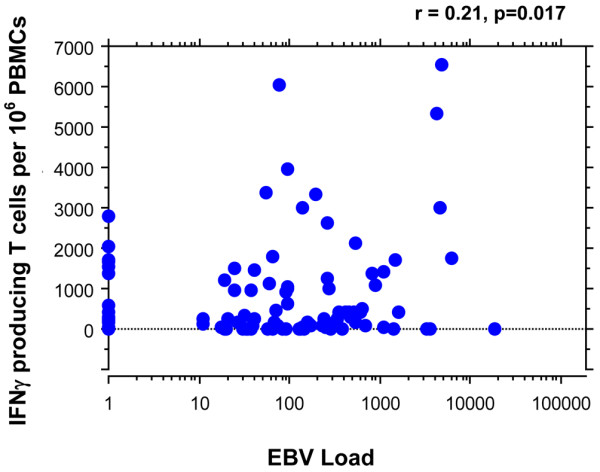
Correlation between the Epstein-Barr virus viral load and Epstein-Barr virus-specific T-cell response. Correlation between the number of IFNγ-producing T cells and the Epstein-Barr virus (EBV) viral load. EBV-specific IFNγ-producing T cells were pulsed with the full set of peptides (latent-cycle peptides and lytic-cycle peptides). PBMCs, peripheral blood mononuclear cells.

For correlation studies between the EBV viral load and EBV-specific T-cell response, 113 patients were studied (66 at week 0 and 47 at week 12). We found a positive correlation between the EBV viral load and the number of EBV-specific IFN-γ-producing T cells in response to the full set of peptides (n = 113, *P *= 0.017, *R *= 0.21) (Figure 4), to latent-cycle peptides (*P *= 0.035, *R *= 0.16) and to lytic-cycle peptides (*P *= 0.011, *R *= 0.16) (data not shown).

#### Unadapted Epstein-Barr virus-specific T-cell IFNγ production under treatment

**Figure 5:**
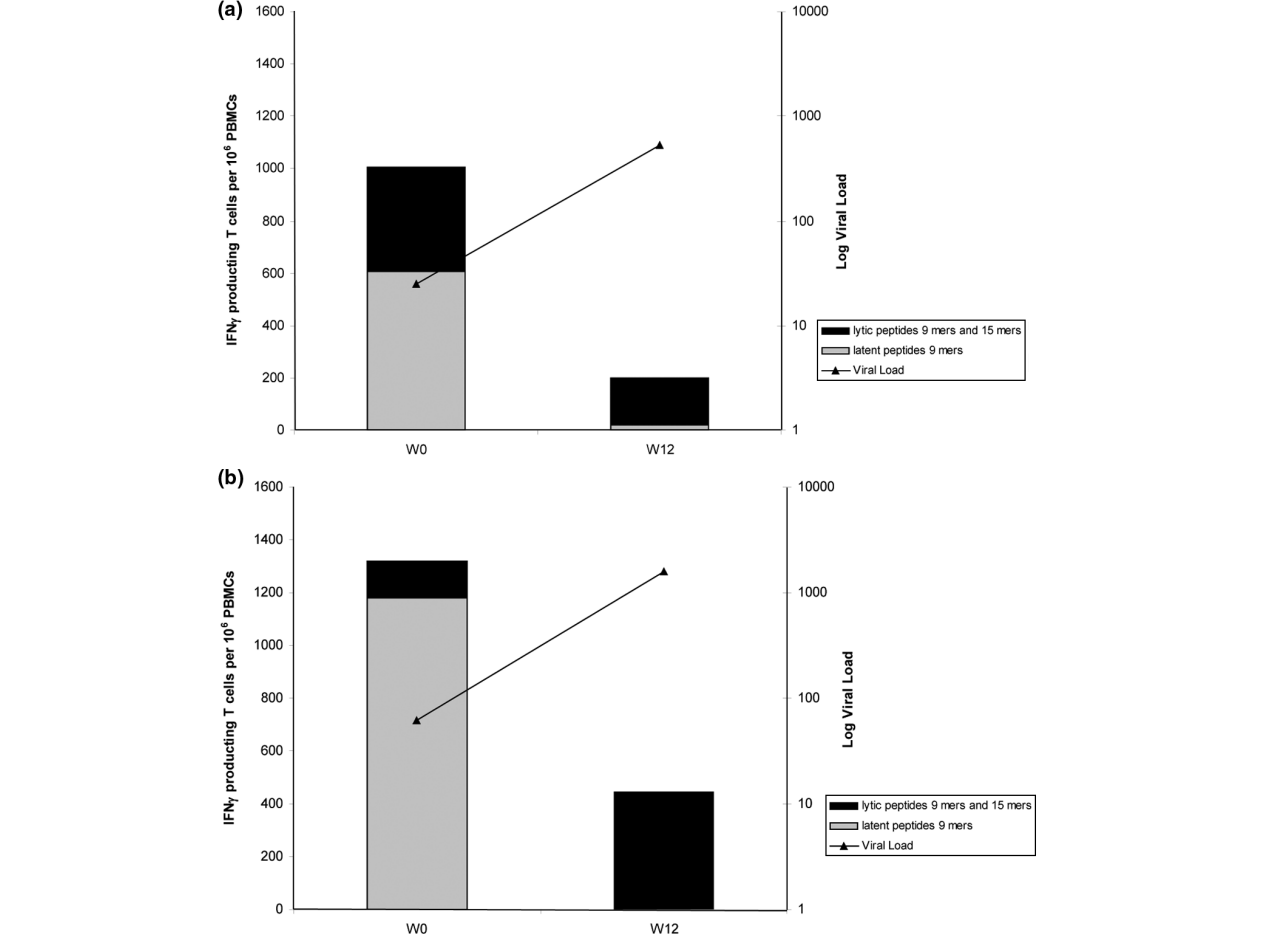
Unadapted Epstein-Barr virus-specific T-cell IFNγ production under treatment. Patients with inappropriate Epstein-Barr virus (EBV)-specific T-cell IFNγ production in response to high EBV viral load under treatment. **(a) **Spondylarthropathy patient with infliximab + methotrexate. **(b) **Rheumatoid arthritis patient with adalimumab + methotrexate. Both responses to latent peptides and lytic peptides are represented. PBMCs, peripheral blood mononuclear cells; W0, week 0; W12, week 12.

Five patients demonstrated inappropriate EBV-specific T-cell IFNγ production (<100 IFNγ secreted T cells and >1,000 EBV copies per 10^6 ^PBMCs). Three of these patients had no or very low IFNγ secreted T cells at week 0 and week 12. Two other patients had an accurate *in vitro *effector function at week 0 but a large decrease of EBV-specific IFNγ secreted T cell number at week 12 despite a concomitant increased level of EBV copy numbers above 1,000 copies per 10^6 ^PBMCs (Figure 5a, b). These two patients were treated with anti-TNF monoclonal antibody associated with MTX: one SpA patient with infliximab, and one RA patient with adalimumab. For both patients, an EBV-specific T-cell response to latent peptides was detectable at baseline but was not detectable at week 12. Nevertheless, the response to lytic peptides was persistent in both cases at week 12.

## Discussion

The present large cross-sectional and longitudinal study showed no abnormality in EBV viral load or EBV-specific T-cell response in patients with RA or SpA at baseline or after treatment with MTX or anti-TNF drugs.

In contrast to other studies [23,24], we did not find increased EBV viral load in PBMCs of patients with RA or SpA. A casual high EBV DNA prevalence in our control group (80%) could account for the differing results, and/or the highly sensitive PCR used in our study might explain such differences. Our cross-sectional study revealed no significant differences between patients and control individuals in the proportion of subjects with positive EBV-specific IFNγ-producing T cells in PBMCs, the mean number of SFCs or the SFC distribution. Two studies have assessed the EBV-specific T-cell response in PBMCs of RA patients [25,26]. The first study found no difference between 49 RA patients and 26 control individuals in the frequency of T cells directed against two immunodominant EBV peptides, but did observe a reduced ability to produce INFγ in RA patients; the effect of immunosuppressive treatment was not assessed [25]. In the second study, EBV-specific effector CD8 T cells were higher in number in RA patients (n = 25) than in control individuals (n = 20), but this study concerned a low number of patients and was only cross-sectional [26]. Actually, this increase in IFNγ secreted T cells was related to increased viral load, which we did not observe in our study.

Our longitudinal results did not reveal any influence of immunosuppressive treatment on the EBV viral load. These results are in accordance with several studies on Crohn disease or RA patients assessing the evolution of EBV viral load under immunosuppressive treatment [27-29]. To the best of our knowledge, no published study has specifically evaluated the longitudinal effect of MTX and anti-TNF drug on EBV-specific T-cell effector functions in patients with RA or SpA. At 3-month follow-up, neither MTX treatment in RA patients nor anti-TNF therapy in RA and/or SpA patients modified these effector functions, regardless of the EBV peptide used for pulsing – latent-cycle peptides or lytic-cycle peptides or the full set of peptides. The lack of increase in the EBV viral load during the same period in all groups of patients agrees with the preserved specific T-cell effector function, which was confirmed by a global correlation between EBV viral load and EBV-specific T-cell response.

Interestingly, in five patients treated with anti-TNF, an inadequate *in vitro *EBV-specific IFNγ production was observed after specific pulse with EBV peptides despite an *in vivo *high viral load. Among those patients, two different profiles were observed. The first profile corresponded to patients without any IFNγ production in spite of high EBV viral loads (>1,000/10^6 ^PBMCs), both at week 0 and week 12. In such cases, the lack of adequate HLA for presenting one of the EBV peptides probably accounted for the absence of IFNγ production after specific pulse. The second profile corresponded to two patients having EBV-specific T-cell IFNγ production at baseline but a discordant evolution between an IFNγ secreted T-cell decrease and an EBV viral load increase after 12 weeks of treatment. In these two patients, immunosuppressive therapy might have impaired EBV-specific T-cell effector functions leading to the lack of control of the EBV viral load. These two patients having been treated with the association of anti-TNF antibody and MTX makes it impossible to differentiate a possible effect of one drug individually. Nevertheless, we never saw profound discrepancies, such as those observed in a pediatric sample in whom post-transplant lymphoproliferative disorder developed after liver transplantation [30]. Since we analyzed data only 12 weeks after the introduction of immunosuppressive treatment, however, we cannot exclude that MTX or anti-TNF therapy could induce impaired EBV control after longer-term treatment. This relative short time duration of immunosuppressive treatment exposure might be considered as a limitation of our study. Nevertheless, post-transplant lymphoproliferative diseases occurring in children, for example, have been reported to occur after a short-term exposure to immunosuppressive treatments (median delay of 12 weeks, range 6 to 56 weeks) [30]. Moreover, with the same methodology (ELISPOT assay), we detected a significant decrease of the specific anti-tuberculosis T-cell response in patients after 12 weeks of anti-TNF therapy [31]. Lastly, in the present study, patients treated with MTX were treated for several years on average, and their results were no different from the MTX naïve patients at baseline.

## Conclusions

In patients with RA or SpA, short-term (3-month) exposure to MTX or anti-TNF therapy does not alter the EBV viral load or the EBV-specific T-cell response. These findings are rather reassuring in light of a suggested increased risk of EBV-associated lymphoma in patients receiving immunosuppressive therapy. Long-term follow-up of the EBV-specific T-cell response, however, is necessary. Moreover, control of EBV is only one mechanism of control of lymphomagenesis and the different epidemiologic studies currently available do not eliminate the possibility of increased risk of non-EBV-associated lymphoma in patients receiving immunosuppressive therapy.

## Abbreviations

bp: base pairs; DMARD: disease-modifying anti-rheumatic drug; EBV: Epstein-Barr virus; FCS: fetal calf serum; HLA: human leukocyte antigen; IFN: interferon; MTX: methotrexate; PBMC: peripheral blood mononuclear cell; PCR: polymerase chain reaction; RA: rheumatoid arthritis; SpA: spondylarthropathy; SFC: spot-forming cell; TNF: tumor necrosis factor.

## Competing interests

The authors declare that they have no competing interests.

## Authors' contributions

XM was responsible for the study design, manuscript preparation and interpretation of the data. CM-R was responsible for sample blood collection, manuscript preparation, interpretation of data and statistical analyses. NG performed the ELISPOT assays and statistical analyses. CA performed the EBV quantitative PCR. JS, MI and SP contributed to the blood sample collection. AU and IG contributed to the ELISPOT assay analyses. GC and AV contributed to interpretation of the data.
